# Malposition of Percutaneous Endoscopic Gastrostomy (PEG) Tube Through the Transverse Colon: A Novel Approach to Conservative Management

**DOI:** 10.7759/cureus.63908

**Published:** 2024-07-05

**Authors:** Zachary A Blashinsky, Joel A Calafell

**Affiliations:** 1 Surgery, Herbert Wertheim College of Medicine, Florida International University, Miami, USA; 2 General Surgery, Baptist Health South Florida, Miami, USA

**Keywords:** new surgical technique, general surgery, peg complication, peg dislodgment, peg tube insertion

## Abstract

Percutaneous endoscopic gastrostomy (PEG) is a common technique for enteral nutrition support. Complications range from skin injuries and leakage to more severe intraabdominal pathologies. This case report describes a patient with invasive right lateral pharyngeal wall squamous cell carcinoma who developed a gastrocolocutaneous fistula following PEG tube malpositioning in the transverse colon performed at an outside institution. Based on the patient’s comorbidities and the associated high-risk nature of the surgery, a transverse colectomy and partial gastrectomy to resect the malpositioned tube followed by a new PEG tube was deemed invasive and would likely have a poor clinical outcome. Instead, the surgeon performed a laparoscopic-assisted PEG tube insertion in another portion of the stomach. The fistulous tract of the original PEG tube was completely sealed and fell out one week following surgery. The patient tolerated feeds through the new PEG tube site. Gastrocolocutaneous fistulas are rare complications of PEG tube insertion with a poorly understood pathophysiology. Here, we analyze the root cause of this condition, steps to mitigate it, and a proposed novel surgical approach for its conservative management.

## Introduction

Enteral feedings have demonstrated superior patient outcomes compared to parenteral nutrition, decreasing costs and reducing hospital-acquired infections [1,2]. First explained in 1980, percutaneous endoscopic gastrostomy (PEG) has become a commonly utilized technique for enteral nutrition support [3,4]. While considered a generally safe procedure, complications, including skin maceration, leakage, and infection, have been reported to varying degrees [2,5,6]. More severe injuries, including intraabdominal bleeding and injury to surrounding organ structures, are concerns that must be mitigated by the performing physician [7-10]. Positioning the transverse colon over the anterior gastric wall could lead to colonic injury during PEG placement [11]. As per protocol in most hospitals, a pre-procedure CT scan of the abdomen is usually done to evaluate the position of the transverse colon. Sometimes, laparoscopic assistance is done to assist in the safe placement of the PEG tube. Gastrocolocutaneous fistula is an epithelial connection between the stomach mucosa, colon, and abdominal skin. Potential etiologies include penetration of the colon during PEG insertion from the skin into the stomach through accidental puncture or gradual erosion of the tube into the adjacent bowel [12]. Here, we report on a novel approach to the management of a patient who developed a gastrocolocutaneous fistula from PEG insertion at an outside institution.

## Case presentation

The patient is an 84-year-old male who initially presented with a pharyngeal squamous cell carcinoma. The patient reported dysphagia and odynophagia for four months and a 40 lb weight loss in one year. The patient had a past medical history of chronic obstructive pulmonary disease (COPD), hypertension, hypercholesterolemia, sleep apnea, and esophageal reflux. Past surgical history was pertinent for a PEG tube insertion for nutrition placed one month prior to presentation at an outside hospital just before the initiation of radiation. The patient was a 50+ year smoker and had quit six months prior to presentation. Computed tomography (CT) one month prior revealed a 3 x 2 x 2.2 x 1.5 cm lesion of the right posterior lateral pharynx. The patient underwent a direct laryngoscopy with biopsy that revealed invasive squamous cell carcinoma positive for P40 and P63, with human papilloma virus (HPV) testing negative. The diagnosis was a right lateral pharyngeal wall squamous cell carcinoma, p16, greater than 2 cm, and had spread to more than one lymph node, stage T2N2cM0. A positron emission tomography (PET) scan revealed intense fluorodeoxyglucose (FDG) avid uptake, showing the right posterolateral oropharyngeal mass extending to the right epiglottic region and being compatible with primary head and neck malignancy. FDG avid uptake of the right and left cervical lymph nodes was compatible with regional metastasis. Flexible nasopharyngolaryngoscopy revealed a pedunculated exophytic lesion emanating from the right pharyngeal wall just above the vallecula, sparing the epiglottis and right aryepiglottic fold, nonobstructive to the airway. Suspected anterior cervical osteophytes from C4-C7 were appreciated as the suspected cause of the esophageal obstruction.

Two months later, it was recommended he undergo a non-surgical approach with radiation therapy as a single treatment modality. Chemotherapy as a radiosensitizing agent was not recommended due to the patient's age and frailty. Approximately three weeks following this recommendation, a cervical C4-C7 anterior osteophytectomy for decompression of the esophagus was performed. The patient was discharged two days later.

**Figure 1:**
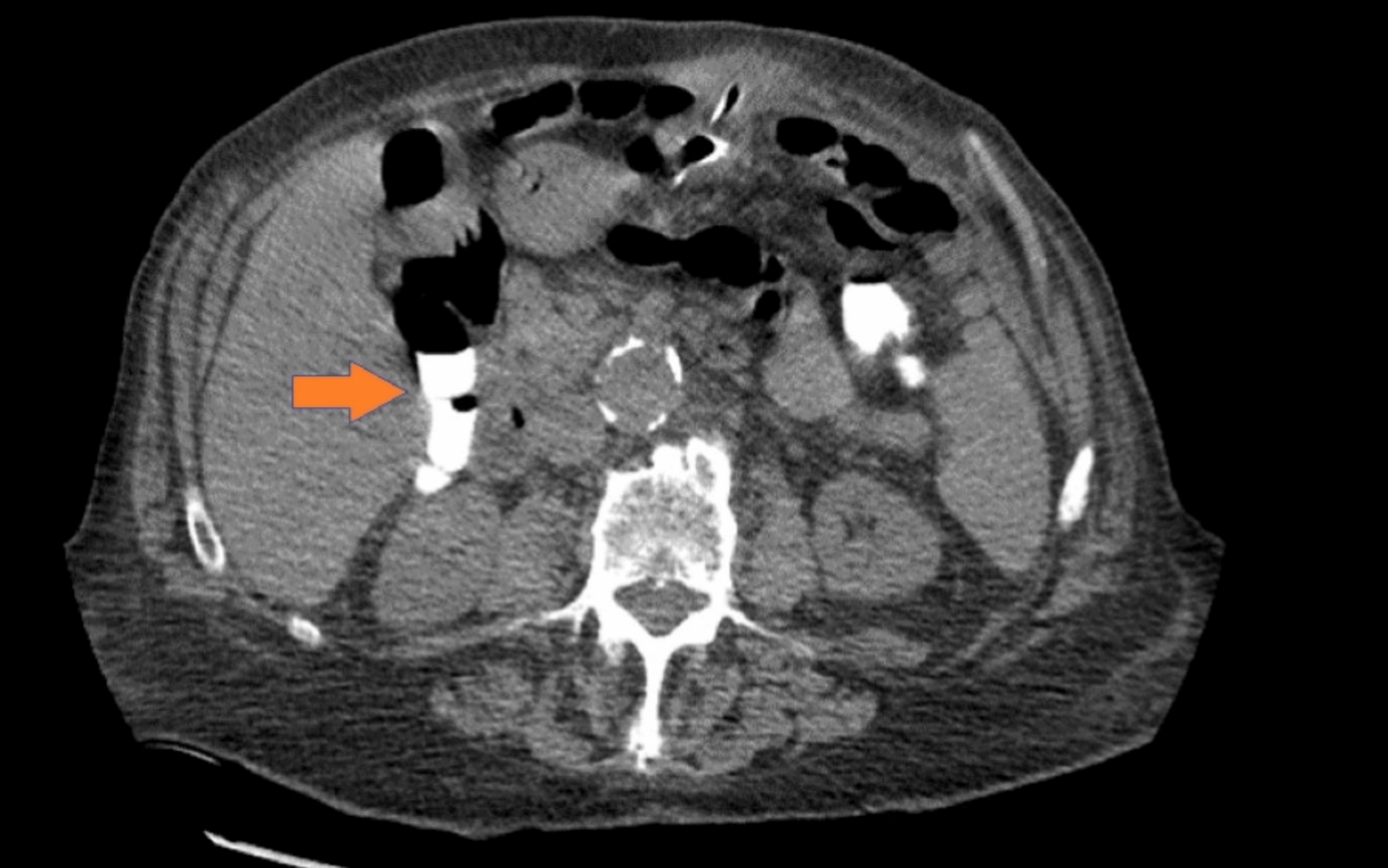
CT scan of abdomen and pelvis with contrast through the PEG tube showing contrast in the colon. PEG: Percutaneous endoscopic gastrostomy

One day after discharge, the patient presented with pain at the PEG tube site and leakage accompanied by multiple episodes of diarrhea on the reinitiation of tube feeds. The patient reported sudden abdominal pain surrounding the gastrostomy tube site upon awakening from surgery. Abdominal CT with contrast injected through the percutaneous enteric tube showed opacification of the transverse colon, indicating improper positioning of the tube within the transverse colon (Figure 1). An upper gastrointestinal (GI) series with water-soluble contrast showed improper placement of the gastrostomy catheter within the transverse colon. It was suspected that the needle and wire for the PEG tube placement penetrated the transverse colon into the stomach, which appeared to be working well, and the patient tolerated tube feeds. However, during his most recent surgery, it is possible that the PEG tube could have migrated out and was in the transverse colon.

The patient and the surgeon discussed surgical options. The most aggressive option was to perform a transverse colectomy and partial gastrectomy to resect the portion containing the tube track and a new gastrostomy tube. However, anastomosis of the transverse colon would be high risk given the patient's comorbidities and his inability to tolerate a mechanical bowel prep. Based on these concerns, the patient and the surgeon agreed that a more nuanced approach would be most appropriate.

The surgical plan was to enter the patient's abdomen laparoscopically and, assuming a well-healed fistula with no signs of infection, perform a lap-assisted PEG tube placement in another portion of the stomach. The original tube would remain in placement and then be removed after several weeks similar to the usual procedure for removing a cecostomy tube.

The pre-operative diagnosis was a malpositioned gastrostomy tube in the transverse colon. The patient was placed under general anesthesia with 60 cc of 0.25% Marcaine mixed with 1% lidocaine with epinephrine given via tap block laparoscopically. The patient was secured on the operating table with both arms tucked, and the abdomen was prepped with betadine, including the previous gastrostomy tube. An infraumbilical incision with a 15-blade scalpel was made, and a Hassan technique was used to dissect the abdominal fascia that was elevated and cut with a 15-blade. The abdominal cavity was visualized, a 12mm trocar was placed, and the abdomen was insufflated to 10 mmHg.

**Figure 2:**
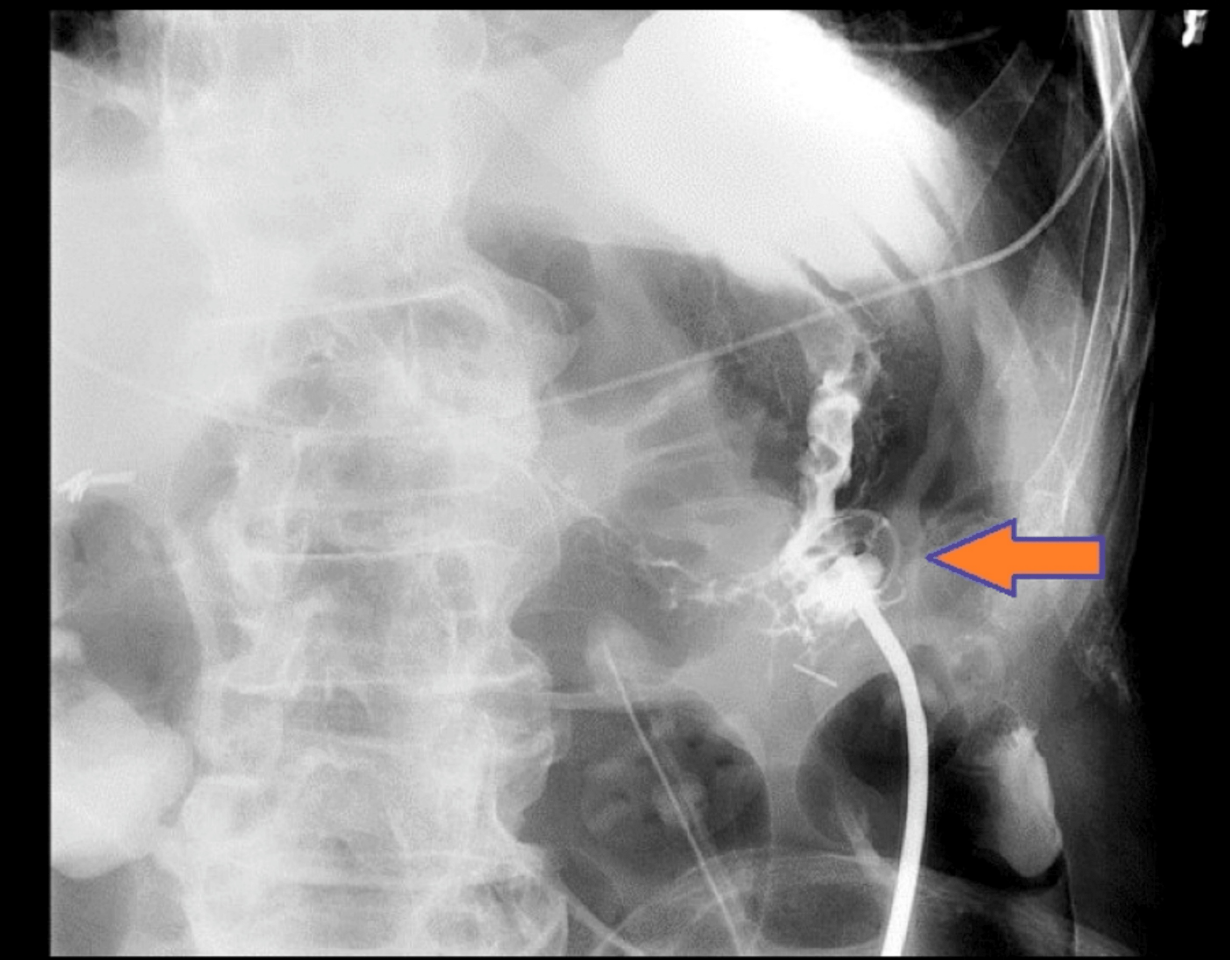
Upper gastrointestinal series (UGI) showing correct placement of new gastrostomy tube.

The 5mm 30-degree laparoscopic camera was inserted and visualized a dilated bowel with a fistulous connection from the previous tube to the transverse colon. The fistulous tract was completely sealed with no signs of any leakage. Then, the camera looked over the colon to visualize a mid and upper stomach window, placing a 5mm trocar in the left lower quadrant for better visualization. The patient’s head and neck surgeon then easily inserted and performed an esophagogastroduodenoscopy, which could insufflate the stomach and create a 5mm incision with a 15-blade scalpel. The tube was placed using the usual “pull” technique. The pneumoperitoneum was then evacuated, and the tube was secured at 2.5cm on the tube from the skin. At three points, this was secured with a 2-0 nylon suture on the bumper. The fascia of the infraumbilical incision was then closed with 0-PDS suture and irrigated with normal saline, and all the wounds were then closed with 4-0 Monocryl. The gastrostomy tube (G-tube) was tested and had no resistance to the irrigation of normal saline (Figure 2). The abdomen was washed and dried, the previous G-tube was marked, and the new G-tube was marked as the one to use. The G-tube will remain clamped and be covered with 4x4 gauze and an abdominal gauze pad (ABD pad) with soft cloth surgical tape.

At the two-week post-operative visit, the patient reported feeling well and tolerating tube feeds via the new PEG tube three times per day. He noted an area of erythema and some purulent drainage surrounding the new PEG tube site. The patient stated that the old PEG tube fell out one week prior. The patient endorsed regular bowel movements, nylon sutures were removed, topical antibiotic ointment was recommended, and dry dressing was recommended surrounding the PEG tube. The area was found to be well-healed at the next office visit.

## Discussion

While PEG tube insertion is a safe and efficient way to provide enteral nutrition, complications of varying severities are possible. Gastrocolocutaneous fistula formation is rare in the literature; however, its incidence rate has been reported to be as high as 2-3% [13,14]. Risk factors for developing gastrocolocutaneous fistulas include insufficient endoscopic insufflation, adhesions secondary to previous abdominal surgery, patient selection, or poor transillumination [12].

The pathophysiology of these fistulous formations appears to be due to the level of insufflation used during PEG tube insertion [5,14-17]. The transverse colon lies anterior to the gastric wall and displaces inferiorly during insufflation. Khattak et al. proposed that an under-inflated stomach may limit the displacement of the transverse colon and increase the likelihood of puncture on PEG insertion [14]. Additionally, an over-inflated stomach may cause excess gas to enter the small bowel, dilating and elevating the colon. The transverse colon could be recognized as a dark band or shadow when illuminating the anterior wall of the stomach, signifying that performing physicians should avoid this structure or change insufflation levels accordingly [18]. Croaker et al. suggested that while insufflation causes anterior rotation of the greater curvature, thereby translating the gastrocolic omentum and colon, excessive insufflation may lead to exaggerated rotation of the typically inferior transverse colon and increase the likelihood of penetration [16]. Patwardhan et al. added to the pathophysiology by explaining that patients with a history of spinal deformity have abnormal posture and positioning of the stomach [17,19]. This increases penetration and fistula formation in patients with similar medical histories, as with our patient.

Patient presentation and treatment modalities varied across the literature. Viso Vidal et al. reported on a gastrocolocutaneous fistula without pneumoperitoneum on CT seven months after PEG tube insertion in a 52-year-old male with Wernicke Korsakoff encephalopathy [20]. The patient presented complaining of stool drainage through the stoma. A colonoscopy showed the balloon in the transverse colon. The balloon was deflated and removed through a cutaneous orifice. In this patient, complete wall defect closure was accomplished using an Ovesco clip rather than spontaneous fistula closure. Hwang et al. repaired the gastrocolocutaneous fistula through an endoscopic suture using hemo-clips at the transverse colon during colonoscopy and at the gastric side with a detachable snare for concrete suture [21].

In the cases described in the literature, many patients appeared to tolerate fistulas for months before the onset of symptoms indicating fistulas. Onset appeared to occur after the replacement of PEG or any activity that caused movement or repositioning of the PEG [15]. Our patient did not present with symptoms until six months after insertion but only one day after surgery, indicating that the fragile fistula formation was disrupted during the procedure or in the pre- and post-operative recovery periods in which the patient was being physically manipulated and transported. A 2007 case series from Israel and a subsequent literature review identified six patients in their hospital with misplaced PEG tubes in the colon [13]. A literature review identified an additional 22 patients. Of those, eight had previous abdominal pathology, 17 developed symptoms after tube replacement, and in 11, the tube had not been changed. Regarding symptoms, 14 reported diarrhea, and 11 reported fecal discharge in or around the tube. Thirteen showed colocutaneous fistula without residual connection to the stomach. A study from Portugal showed three patients with fistula formation, with all three reporting problems after their first PEG replacement at approximately six months after original PEG insertion [22]. Yamazaki et al. reported on 11 cases, five of which had previous history of abdominal surgery [23]. While the length of time from insertion and onset of problems differed significantly, between a few days to a year, four patients had removal of the PEG as treatment, whereas five had surgical gastrotomy. One had a laparotomy. Our patient did not require such invasive treatment as the fistula spontaneously closed, the original PEG fell out, and no issues occurred after the new PEG was inserted. Our patient’s symptoms did not begin until after ENT surgery. The gastrocolocutaneous fistula was likely disturbed during surgery or the transfer process, causing the PEG to lose its connection with the stomach and insert directly into the transverse colon, initiating his symptoms of diarrhea, pain, and discharge. Our patient appeared to have discharge but not stool; likely, the discharge was from attempted feedings.

Prevention mainly focuses on proper transillumination through the abdominal wall [5,18,24]. Strodel et al. suggest using a syringe to aspirate the needle when inserting it through the abdominal wall. If air is appreciated before visualization of the needle in the stomach, then the needle has likely punctured bowel located between the anterior gastric wall and cutaneous surface [24]. Our patient did not possess the operative report from his original PEG tube insertion, which prevented us from theorizing the etiology of the fistula complication.

## Conclusions

Here, we describe a conservative approach to managing the malposition of PEG tube insertion with gastrocolocutaneous fistulas. More data is needed to better understand the pathophysiology of penetration of the transverse colon in PEG insertion. More research must be conducted to determine a standard therapeutic approach to managing PEG malpositioning with concomitant gastrocolocutaneous fistula. Performing physicians must be mindful of this potential, albeit rare, complication and recognize the similar symptoms reported across the limited literature. Understanding this procedural complication may assist clinicians when treating patients with similar medical histories and abnormal presentations.
